# Supplementary material for: Effects of intra-articular Platelet-Rich Plasma (PRP) injections on osteoarthritis in the thumb basal joint and scaphoidtrapeziotrapezoidal joint
Source: PLoS One. 2022 Mar 8;17(3):e0264203. doi: 10.1371/journal.pone.0264203 (PMC8903265; doi:10.1371/journal.pone.0264203)
Supplement: Attachment — Submitted filename: Response to review PLOSone.docx [file pone.0264203.s002.docx]

**Response to reviewers**

Thank you for a valuable review. Below we address the reviewers’ concerns point by point.

*Reviewer #1:*

*I focus on the statistical aspects for the study. Overall, I find the analysis relatively simple which can be particularly problematic when the sample size is small.*

We have addressed the reviewer’s concern about too simple statistical analysis and with help from a biomedical statistician, we have remade the analysis with a GEE model. This made a significant difference since the formerly significant improvement of pain was no longer significant after the confounding predictors were considered in the analysis. Hence, the conclusion and a major part of the discussion is revised.

*Some particular concerns are*

*a) There was mention about excluding patients from the study because they had incomplete data. It is not clear what exclusion rule was used whether the missingness had something to do with the disease progression or the pain the patient is suffering. Are data missing at random or completely missing at random or otherwise? Statistical methods will then have to specially used for different types of missingness;*

Four patients had to be excluded since they did not attend the 3 months assessment. We now write this more clearly in line 62-64. The former wording was unclear. The reason for not attending could not be found in the medical records. We consider them missing at random. After ha have redone the analyses with correction for predictors (please see below), a potential difference in the excluded patient compared to the analysed sample, should not be a problem.

*b) There are statistical tests that are more reliable for analysis small sample data that are more reliable; permutation tests may be better alternatives. Similarly, Shapiro-Wilk’s test results on whether the data are normally distributed can also be questionable for small data set;*

We now use GEE for analyses instead so this should not be an issue.

*and*

c*) There were demographic data or covariates on the patients but there was no discussion if some of them play a role in explaining the findings the authors obtained.*

The covariates/possible predictors are now included in a GEE model.

*Reviewer #2:*

Well written and pleasant to read paper.

Introduction:
*- How is it relevant to only focus on the short-term effects of this treatment? I'm not sure it is, but please explain. As CMC1 OA is a chronic condition (we will all get osteoarthritis of the thumb if we live long enough), we should focus on treatments that have long-term benefits. Most likely a "quick fix" with an injection or surgical intervention is not the solution.*

We believe that the short-term aspect is relevant since injections are assumed to foremost have a short-term effect. We did not have long-term data and we agree that the short-term follow-up is a limitation and mention this in the discussion line 173-174.

*- Please change "conservative treatment" to "non-operative treatment".*

This is now changed.

Methods:
*- The 3-4 weeks interval between the injections: please explain how you chose this time interval.*

There is no consensus or scientific base for the number of PRP injections or the optimal interval. The patients had injections with 3-4 weeks based on recommendations from Arthrex. Due to the retrospective design this was not part of the study design.

*- Symptom intensity of CMC1 OA is strongly related to the influence of psychosocial aspects; patients with greater symptoms of catastrophic thinking and depression have greater pain intensity. In many multivariable analysis, these are the strongest contributors to several outcomes including pain (see papers of Becker et al., Wilkens et al., or Prof. Ring). At least, this must be mentioned as it could lead to a potential bias in the findings of the current paper*.

We agree with the reviewer and we have no included high pre-injection pain as a predictor in the analysis. The issue is also discussed in line 162-166. Although the above mentioned authors have produced many interesting articles, we could not find a suitable reference from these specific authors for this particular issue.

*- How did you estimate an SD of 2 points for NRS pain?*

SD 2 was estimated based on patient-rated pain in a large sample of patients planned for surgery for thumbbase osteoarthritis (Wilcke et al.) with a mean pain on load of 76 (SD 17) of 100 and the small study of Loibl et al; pain VAS 4.0 (SD 2.4) at 3 months after a PRP injection. This is now stated more clearly in line 76-77. In retrospect, the estimation was a bit low since the SD (which we do not present in the manuscript that reports median and IQR) of pain on load after 3 months were 3 corresponding to a required sample of 20 so we don’t think the study is underpowered.

*- What where the inclusion criteria or selection criteria for patients to receive a PRP injection?*

The selection criteria were osteoarthritis in the thumbbase or STT-joint. This is now pointed out in line 51.

*Results & Discussion:
- Please write out "thirty-three" and other numbers that are at the beginning of a sentence.*

This is now corrected.

*- Line 111-112: these results don't exceed the MCID. Although there is some variety among studies about the MCID for pain scores (NRS/VAS), most agree that an improvement of 1 point is not enough to reach an MCID in outcome (Danoff 2018, Lee 2003). Even the study referred to in this manuscript (Salaffi et al.) supports the use of a "much better" improvement on pain relief as a clinically important outcome: and that is 2 points. I think the authors must revise this point and their conclusion.*

According to reviewer #1:s concern about analyses being too simple, we have now remade them I cooperation with a statistician using a GEE model for longitudinal data with the possibility to correct for possible predictors. Interesting, this analysis showed no significant impact of PRP injection. Hence the conclusion and discussion are quite extensively revised.
