# Supplementary material for: Effects of intra-articular Platelet-Rich Plasma (PRP) injections on osteoarthritis in the thumb basal joint and scaphoidtrapeziotrapezoidal joint
Source: PLoS One. 2022 Mar 8;17(3):e0264203. doi: 10.1371/journal.pone.0264203 (PMC8903265; doi:10.1371/journal.pone.0264203)
Supplement: Attachment — Submitted filename: Rebuttal letter 2 PLOSone.docx [file pone.0264203.s003.docx]

Response to reviewers

Reviewer #2: I would recommend to accept this paper. However, one more suggestion:

* The conclusion of this paper is that PRP injections do not lead to pain relief, decrease of physical limitations or improvement of strength. The last paragraph of the discussion however, seems like a plea for the harmless use of PRP injections. Please look at this again. And delete the last sentence: "Although this study has methodological flaws, and it is important to test PRP in randomized placebo-controlled studies”, our study results suggest that PRP injections have no effect on pain intensity, physical limitations, or thumb strength among patients with osteoarthritis in the thumb basal joint or STT-joint."

**According to the reviewer’s suggestion, we have deleted the last sentence “Although this study has methodological flaws, and it is important to test PRP in randomized placebo-controlled studies.”**

Reviewer #3: Thank you for the opportunity to review this paper.

I credit the authors for prospective data collection. However, I cannot see why the authors did not define clear study objective/question and a study protocol a priori in order to collect a prospective case series. This leads to several shortcomings.

**We agree with the reviewer that a prospective case-series would have been preferable. However, this is a retrospective study based on medical protocols.**

Perhaps the biggest concern is the lack of explicit inclusion and/or exclusion criteria which leads to major heterogeneity between participants: Both thumb basal joint and STT arthrosis (in my opinion, these conditions may be related to different biomechanical changes), age 34-86, posttraumatic or primary arthrosis (?), the duration of symptoms before treatment, pre-treatment pain intensity (no borders for inclusion/exclusion), associated conditions that might affect the result (e.g. small joint arthritis in general, thumb MCP arthritis), previous trauma or surgery of the hand/wrist and treatment of contralateral TMC/STT joint arthritis (successful non-operative/operative treatment of contralateral TMC/STT might affect patients expectations).

**We agree that this is a weakness with the retrospective design as discussed in line 169-176. On the other hand, the wide inclusion criteria make the result generalizable and there is no comparison between groups that would be biased by the heterogeneity.**

While the authors have assessed the severity of the arthritic changes - in my opinion it is as important to assess the joint congruency as the thumb basal joint might be clinically stable, instable or chronically subluxated - which might affect the outcome of the injection as the biomechanical basis of the disease might be different.

**This is an interesting point. However, since joint stability in thumb base osteoarthritis was not a variable recorded in the medical records, the effect if the joint stability could not be assessed.**

Another concern lies in the preparation of PRP - how rich is platelet-rich? No laboratory testing is made to control for the quality of the agent.

**Testing the concentration is not possible given the retrospective design. This is a pragmatic study of the effect of PRP prepared with a commercial kit. As the reviewer hints, the concentration may theoretically affect the effect of PRP, and this is discussed in line 132-34.**

Moreover, how do the authors believe that the quantity (0,5-2ml) of PRP affects the outcome?

**We have not included injected quantity as a covariate in the analysis since two different joints with different sizes (CMC1 and STT) were treated and we believe that quantity would be difficult to assess as a covariate. Therefore, we cannot say anything about the potential effect of the injected quantity.**

How was the interval between treatments decided? The regulatory aspects of the use of PRP for TMC and STT OA should also be declared.

**The interval of interval of 3-4 weeks was recommended by the manufacturer (Artrex). This is now stated in line 52. There are no regulatory aspects of PRP as discussed in line 180-1.**

The enrollment lasted from sep 2018 to sep 2019. Was it a consecutive series? Was the enrollment based on patient preference? How many patients declined?

**As described in line 45-48, all patients that received PRP treatment for radiologically and clinically verified osteoarthritis in the thumb basal joint or STT-joint between September 1st2018 and September 30th2019 were included. This is not a prospective study and hence, there was no invitation to participate in a study.**

Were there any exclusion criteria?

**Exclusion criteria was lack of data as describes in line 62-63 “Patients that did not attend the assessment after four months were excluded due to lack of data.“. We have added the sentence “There were no other exclusion criteria.” To clarify this.**

Post-treatment regimen? The authors state that “The patients could resume work and daily activities immediately after the injection as tolerated.” - did the patients use a splint?

**We have no information of whether and to what extent the patients used splints. This is now mentioned as a weakness in the discussion line 169-73.**

Was there any hand exercise regimen? The authors report that two patients were prescribed pain medication at first injection, whereas 20 did not use any pain medication. Seven patients used pain medication for “unrelated reasons” All of these might significantly affect the outcome as the post-treatment protocol was not defined.

**There was no post-injection hand exercise protocol. This is now stated in line 55-56. The use of pain killers was included as a possible predictor in the model. We have added the sentence “Manually demanding work, affected joint, and use of analgesic did not affect the outcome variables.” in line 109-110 to clarify that these included predictors in the model did not have an effect.**

In the statistics paragraph the authors have presented a power calculation. A post-hoc power calculation seems off, why not just look at 95% CI? Why not repeated measures analysis of variance? **The power calculation was performed prior to the analysis to estimate if there were enough available patients to enable a meaningful evaluation of this sample.**

**GEE is more efficient and flexible than Repeated measures ANOVA. GEE is an extension of generalized linear models such as RM-ANOVA to cover repeated measures and other forms of dependent data and can handle categorical outcome; non-normal and non-linear data.**

The results are very brief and mainly presented without numbers (percentage, SD, IQR, 95% CI) or significance levels.

**Could it be that the reviewer has read the first version and not the revised manuscript? Line 99-109 are all results with numbers; beta coefficient (β) with 95%CI.**

Lines 111-114 are not results per se but patient characteristics (a methodological concern).

**Is this the correct lines or do they refer to the non-revised manuscript? Lines 111-114:**

**“months after the first injection. There were no recorded infections or other adverse events.**

**Discussion**

**We could not find a significant effect on patient-reported pain, hand disability or strength in”**

**In the original, non-revised manuscript line 111-114 was:**

**“22 patients had used a splint prior to the PRP treatment, and 20 patients had previously tried corticosteroid injections. Two patients were prescribed analgesics for pain in the thumb base or STT-joint at/in conjunction with the first injection, 7 patients frequently used analgesics for unrelated causes and 20 patients were not prescribed any analgesics during the study period.”**

**If the reviewer addresses the lines in the original manuscript, we do not really agree. This is a description of the sample and that type of information is usually presented in result and we do not think that this information belongs under Methods.**

The minimally clinically important difference for pain presented in power calculation is 2 points (2 SD). However, the authors interpret a 1-point decrease in NRS a “substantial improvement”. How is this clinically relevant?

**We do not recognize this from the current manuscript, and we are concerned that the reviewer refers to the original and not the current revised manuscript?**

*Line 72-74: In patients with chronic musculoskeletal pain, a reduction of two points in the NRS is considered as a substantial improvement (“much better”) by the patients and is recommended as a clinically important outcome [15].*

*Line 119-121: However, even when possible confounders were not taken into account, the mean individual reduction in pain NRS did not amount to two points (table 2), corresponding to a substantial improvement [15].*

The patients reported the pain to be “slightly better” - which could represent treatment bias and reflect the expectations of the patients. Patient acceptable symptom state (PASS) would better reflect the clinical outcome as PASS is the highest symptom level at which patients consider themselves well.

**Again, we do not recognize this from the current manuscript, and we are concerned that the reviewer refers to the original and not the revised manuscript?**

**PASS seems like a good and relevant concept and a good a complementary measure to MCID but this was not used in the medical records and as to our knowledge there are no published cut-off value for osteoarthritis in the hand/wrist.**

Moreover, the authors report that 15 patients requested a new PRP injection, and two patients wished for a corticosteroid injection at the follow-up 4 months after the first injection. Thus, at least 58% of the patients were probably not happy with the treatment. Was surgery an option? What about the 42%, what happened then? I would be interested if the treatment reduces the number of surgeries in the long-term. As the authors point out in the introduction - successful conservative treatment that delays or reduces the need for surgery is of great value. However, the present paper does not give any clinically relevant information on the matter as the results are very short term.

**We cannot provide any information about further treatment including operation. This study does not give any information about the long-term effect in terms om reduced number of operation but only the short-term results as stated in line 5, 41, 117, 176.**

The subject of the present manuscript is very welcome as an increasing interest in biologic agents - as nonoperative treatment modalities or to augment surgical procedures - has been observed in recent years. However, the methodological shortcomings of the present paper make it hard to support the publication of this paper.

**We agree that this study has shortcomings. But at this time when PRP is widely administered despite limited evidence of its effect and reports may be affected by commercial interests, we believe that it is important to publish all available clinical results.**
